# Physical durability of PermaNet 2.0 long-lasting insecticidal nets over three to 32 months of use in Ethiopia

**DOI:** 10.1186/1475-2875-12-242

**Published:** 2013-07-15

**Authors:** Aprielle B Wills, Stephen C Smith, Gedeon Y Anshebo, Patricia M Graves, Tekola Endeshaw, Estifanos B Shargie, Mesele Damte, Teshome Gebre, Aryc W Mosher, Amy E Patterson, Yohannes B Tesema, Frank O Richards, Paul M Emerson

**Affiliations:** 1 The Carter Center, Atlanta, GA, USA; 2 Centers for Disease Control and Prevention, Atlanta, GA, USA; 3 The Carter Center, Addis Ababa, Ethiopia; 4 Emory University, Atlanta, GA, USA; 5 School of Public Health, Tropical Medicine and Rehabilitation Sciences, James Cook University, PO Box 6811, Cairns, Qld, Australia

## Abstract

**Background:**

Ethiopia scaled up net distribution markedly starting in 2006. Information on expected net life under field conditions (physical durability and persistence of insecticidal activity) is needed to improve planning for net replacement. Standardization of physical durability assessment methods is lacking.

**Methods:**

Permanet®2.0 long-lasting insecticidal bed nets (LLINs), available for distribution in early 2007, were collected from households at three time intervals. The number, size and location of holes were recorded for 189 nets used for three to six months from nine sites (2007) and 220 nets used for 14 to 20 months from 11 sites (2008). In 2009, a “finger/fist” sizing method classified holes in 200 nets used for 26 to 32 months from ten sites into small (<2 cm), medium (> = 2 to < =10 cm) and large (>10 cm) sizes. A proportionate hole index based on both hole number and area was derived from these size classifications.

**Results:**

After three to six months, 54.5% (95% CI 47.1-61.7%) of 189 LLINs had at least one hole 0.5 cm (in the longest axis) or larger; mean holes per net was 4.4 (SD 8.4), median was 1.0 (Inter Quartile Range [IQR] 0–5) and median size was 1 cm (IQR 1–2). At 14 to 20 months, 85.5% (95% CI 80.1-89.8%) of 220 nets had at least one hole with mean 29.1 (SD 50.1) and median 12 (IQR 3–36.5) holes per net, and median size of 1 cm (IQR 1–2). At 26 to 32 months, 92.5% of 200 nets had at least one hole with a mean of 62.2 (SD 205.4) and median of 23 (IQR 6–55.5) holes per net. The mean hole index was 24.3, 169.1 and 352.8 at the three time periods respectively. Repairs were rarely observed. The majority of holes were in the lower half of the net walls. The proportion of nets in ‘poor’ condition (hole index >300) increased from 0% at three to six months to 30% at 26 to 32 months.

**Conclusions:**

Net damage began quickly: more than half the nets had holes by three to six months of use, with 40% of holes being larger than 2 cm. Holes continued to accumulate until 92.5% of nets had holes by 26 to 32 months of use. An almost complete lack of repairs shows the need for promoting proper use of nets and repairs, to increase LLIN longevity. Using the hole index, almost one third of the nets were classed as unusable and ineffective after two and a half years of potential use.

## Background

Long-lasting insecticidal bed nets (LLINs) are considered a vital component in the worldwide effort to prevent malaria transmission in malaria-endemic countries [1]. In 2007, the World Health Organization’s Global Malaria Programme recommended immediate scale up of LLIN distribution from national programmes and partners. As a result, large-scale distribution efforts have been launched to meet this goal, particularly in sub-Saharan Africa, by a host of governments, non-governmental organizations and collaborations with local governments and international charitable organizations.

Ethiopia’s impressive scale-up of net distribution started after the development of a new strategic plan in 2005, and resulted in household ownership of at least one net (any type) increasing from 4.5% in 2005 to 72.5% by 2007 [2]. In 2006, LLINs were instituted as one of the primary methods of vector control as they removed the need for regular re-treatment of impregnated nets with insecticide [1]. These “long lasting properties” were tested and standardized by the World Health Organization Pesticide Evaluation Scheme (WHOPES), requiring that LLINs retain effective insecticidal activity for at least 20 laboratory washes and under field conditions for at least three years [3]. Manufacturers’ claims regarding the life expectancy of their products are generally based on the results of these types of studies; in particular the length of time and the number of laboratory washes a net can endure before insecticidal activity is lost. However, most evaluations to date of LLIN performance and durability have been restricted to laboratory tests, experimental hut studies and limited field trials [4,5]. To date, relatively few LLIN evaluation and monitoring studies have addressed net durability after usage in the field. Such real world information can be valuable for determining the validity of and improving the laboratory test methods, aiding future LLIN purchase decisions and determining the timing and strategy of net replacement campaigns. Moreover, such studies may identify ways these products can be further improved and also characterize the influence of specific net user behaviours on net durability.

Previous studies of net durability after real-world use have shown variable results. Smith *et al.*[6] reported on the condition of 255 bed nets collected in one district in Ghana, 38 months after a district-wide distribution campaign. In a subset of 50 polyester (primarily PermaNet®1.0) nets selected for detailed study, a total of 2,023 holes >0.5 cm in diameter were recorded and 31 holes >10 cm in diameter. Most holes were located on the bottom third of the net. Seam failures were seen in 50% of the nets, and a low rate of repair was also observed. A study by Killian *et al.* of PermaNet®1.0 and 2.0 in Uganda revealed that >70% of the nets had holes after one year and >85% had holes after two years [7]. Holes were mostly (75.6%) located on the lower half of the net. Also, the poor physical condition of the nets suggested that many campaign nets may have been worn out and discarded prior to collection, thus skewing the results by removing the unknown number of the most deteriorated nets. For Olyset™ LLINs collected in 2004 after seven years of use in Tanzania, Tami *et al.*[8] found that most (55%) of the nets had six to 15 holes >2 cm. The diversity of measures of net damage used shows that there is a need for standardization of methods for monitoring net durability, as studies conducted to date have not used consistent methods.

The Ethiopian Federal Ministry of Health (FMOH) set an ambitious national goal of full population coverage with a mean of two LLINs per household through distribution of about 20 million LLINs, in malarious areas, by the end of 2007. To contribute to this effort, The Carter Center assisted in procurement and distribution of three million LLINs in selected areas of three regions of Ethiopia: Amhara, Oromia and Southern Nations, Nationalities and Peoples (SNNP) Regions. The data presented here are part of the monitoring process following the large-scale distribution of PermaNet®2.0 LLINs in the three regions, starting in 2007. The aim of the work was to assess the decline in net functionality over time from samples of nets with known time of distribution, as well as establish a simple and repeatable method to assess physical durability and insecticidal activity of LLINs. These data will inform the timing and nature of the future net replacement strategies and may also improve behaviour change communication about net care and lifespan. This paper reports on the findings related to the physical durability.

## Methods

### Collection

The PermaNet®2.0 LLINs distributed during the campaign assisted by The Carter Center were all light blue, 180 × 190 × 150 cm rectangular nets made with 75 denier polyester yarn. They comprised nets from 14 numbered manufacturing batches. During the campaign, each batch was distributed within a different administrative zone, except for one batch that was distributed in both the North Wollo and North Shewa zones of Amhara Regional State. Recording these batch numbers prior to distribution allowed collection teams to identify a net in the field as being from the campaign.

**Figure 1:**
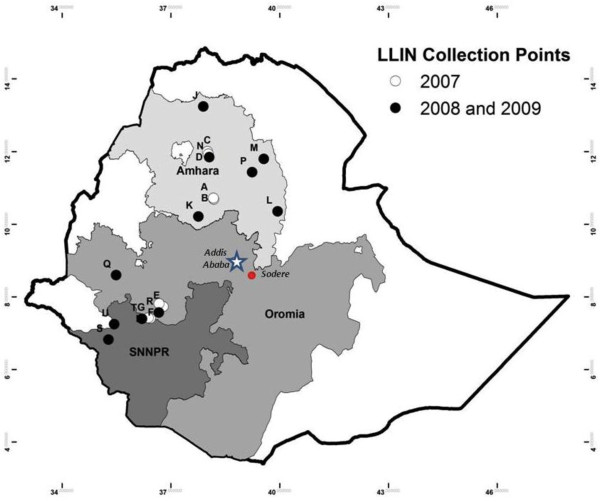
**Long-lasting insecticidal bed nets’ collection areas.** Map of Ethiopia showing location of collection sites. Open circles denote areas of 2007-only collections. Closed circles denote areas where nets were recovered in both 2008 and 2009.

The first LLIN collection was conducted in August 2007 in eight sites (identified by letters A through H) in four zones (see Figure 1). In order to sample from each Regional State, two zones were chosen in Amhara (East Gojjam and South Gondar), and one each in Oromia (Jimma) and SNNPR (Kaffa) regions. For each zone, two *kebeles* (neighbourhoods) within one *woreda* (district) were chosen by convenience sampling from areas that had received nets in batches procured by The Carter Center; the choice was restricted to sites reasonably accessible to vehicles (not more than 30 minutes’ walk from a road). The target was 25 nets per site for a total of 200 nets. It was subsequently determined that some collected nets in Kaffa zone (sites G and H) were not from The Carter Center distribution; they were excluded as their time in use was not known.

The second collection was conducted between November and December 2008, and the third from July to November 2009. Because relatively large variation in net condition between sites had been observed in the first collection in 2007, the number of sampling units in the second and third collections was increased to cover all 11 zones that were assisted by The Carter Center; only one site per zone was selected (sites J through U in Figure 1). For these collections, a list of sites was selected randomly from a complete list of *kebeles* that had received nets from the above-mentioned batches. When a site was deemed inaccessible, the next site in the list was substituted. Twenty nets were collected per site in 2008 and 2009, and during each household visit observers first checked that the net originated from the correct distribution batch numbers.

Within the *kebele*, households were selected for net collection by starting at the first household encountered on entering the *kebele*, followed by immediate neighbouring houses. Household visits were conducted with the co-operation and assistance of the local government health extension office in each *woreda*. One net was collected per household; if there was more than one net available, a net that was hanging was collected with the consent of the head of the household. The visiting team provided a new PermaNet®2.0 replacing the old net. Collected nets were labelled with a unique identifier code and placed individually in plastic bags for storage and transport. The latitude, longitude and elevation were recorded for each household using a GPS receiver (12-Channel Garmin® E Trex™, Garmin International, Olathe, KS, USA). The same *kebeles* were visited in both 2008 and 2009, but different households (the next closest houses to those previously visited in 2008) were selected in 2009.

Nets from the first round of collections were evaluated at the Centers for Disease Control and Prevention (CDC) in Atlanta, USA. Nets from the second and third collections were kept in-country and evaluated at The Carter Center office in Addis Ababa.

### Time period of use

The time that nets had been in use was estimated from the reported time (month) of distribution and the known time of collection. Most nets were distributed between March and June 2007 (three to six months before the first collection in 2007), but there was an exception in site J (Dib Bahir *kebele* in Debark *woreda*, North Gondar zone) where the nets were not distributed until four months before the second collection in August 2008. The nets were therefore not grouped by year of collection for analysis, but by “time of use groups” as follows:

● Group 1 (three to six months of potential use): comprises nets from the eight collection sites (A through H) in 2007 plus nets from site J collected in 2008 – total nine sites and 189 nets;

● Group 2 (14 to 20 months of potential use): comprises nets from the remaining ten collection sites in 2008 (sites K through U) and from site J collected in 2009 – total 11 sites and 220 nets.

● Group 3 (26 to 32 months of potential use): comprises nets from the remaining ten collection sites in 2009 (sites K through U) – total ten sites and 200 nets.

### Physical evaluation

Nets were individually deployed over a rectangular 180 × 180 × 180 cm frame to be examined for holes, seam failures and repairs [6]. In 2007 and 2008 (collection groups 1 and 2, excluding site J in group 2) the size and location of each hole was recorded for each net. Most holes were elliptical, and the hole size was measured as the long axis of the ellipse to the nearest cm. Only holes ≥0.5 cm were counted, and hole sizes of 1 cm and above were rounded to the nearest cm. Hole location was recorded separately for each panel of the net (four sides and the top panel) and the distance in cm from the bottom edge of the net was recorded for the side panels. This method of hole assessment was extremely laborious and time consuming and would not be possible to replicate inside houses where nets are actually hanging. Therefore in 2009 (collection group 3 plus site J in collection group 2) hole size assessment was done by the finger/fist method. Any hole smaller than a finger was classified as “small”, holes between finger and fist size as size “medium”, and holes larger than a fist as size “large”. The finger and fist measurements of the person (average size adult male) doing the measurement were: finger 1.5 cm; fist 10 cm. In order to compare the three collections over time, sizes of holes from the 2007 and 2008 collections were converted to the small/medium/large categories using the cut-offs <2 cm: small; > = 2 to < =10 cm: medium; >10 cm: large.

Seam failures were defined as a hole ≥1 cm caused by a gap between two sides of a stitched seam or corner. Repairs were defined as evidence of hand stitching over a hole, application of a patch, or knotting the fabric to close a hole. No nets in 2007 showed evidence of repair; in 2008 and 2009, location of the observed repairs (distance from the bottom of the net to the centre of the stitch, patch, or knot) was recorded.

### Statistical analysis

The total number of holes per net (total and by size category) and the hole sizes (for 2007 and 2008) were described by the median and interquartile range, and the overall range by net. Comparisons over time group of collection used the Wilcoxon sign rank test. Proportions of nets with holes or repairs were compared over time using the χ^2^ test.

### Hole index

There are two steps to estimating a summary hole index for a net: the first is classifying the holes into size groups (small, medium, large, etc.), and the second step is multiplying the numbers of holes in each of these groups by a factor (estimated from predicted area of an average hole in that category) and summing to reach a summary “proportionate hole index”. The intent of the hole index is to assign appropriate weight to larger holes that may let more mosquitoes in. At the time this study was started there was no consensus on method of quantifying net holes, placing them into categories of size, or estimating a summary index. Since then, two schemes have been proposed:

1. WHO [9] recommended four size categories of 0.5 to 2 cm, 2 to 10 cm, 10 to 25 cm and >25 cm. The midpoint diameter of each is estimated to be 1.25, 6, 17.5 and 30 cm. The relative multiplication factors based on corresponding hole areas for these groups are 1, 23, 196 and 578.

2. Kilian *et al.*[10] and Batisso *et al.*[11] used three size categories <2 cm, 2 to 10 cm and >10 cm (finger, fist and head). They estimated average hole areas to be 4, 36 and 225 sq cm respectively, giving multiplication factors of 1, 9 and 56 for small, medium and large holes to arrive at the proportionate hole index.

Since this study used three hole size categories with approximately the same cut-offs as Kilian *et al.*[10], a proportionate hole index for each net was estimated as follows:

Hole index = [number of small holes < 2 cm + (9 × number of medium holes > = 2 cm to < = 10 cm) + (56 × number of large holes > 10 cm)]. Hole indices per net were averaged for site and time-group summary estimates.

Nets were classified into four levels based on physical condition using the hole index, following Kilian *et al.*[10] and Batisso *et al.*[11]. Nets with hole index <25 were classed as “good”, hole index 25 to 174 “fair”, hole index 175–299 “mediocre” and hole index > =300 “poor”.

## Results

### Net collections

The location of each site is shown on the map in Figure 1. The site elevation, number of nets collected and time period of potential use in each site are shown in Table 1.

**Table 1:** Long-lasting insecticidal bed nets’ collection sites

**Site**	**Year of collection**	**Collection group**	**Time in potential use (months)**	**Region**	**Zone**	**Woreda**	**Kebele**	**Elevation (meters)**	**No. of TCC nets collected**
A	2007	1 (3–6 months)	3	Amhara	E Gojjam	Enarge Enawga	Titar	2566	25
B			3	Amhara	E Gojjam	Enarge Enawga	Dejagamna	2517	25
C			3	Amhara	S Gondar	Farta	Medeb Gubida	1991	25
D			3	Amhara	S Gondar	Farta	Teraroch	2325	25
E			6	Oromia	Jimma	Mana	Gudata Bula	1976	25
F			6	Oromia	Jimma	Mana	Haro	1682	25
G			3	SNNPR	Kaffa	Gimbo	Arboba	1431	5
H			3	SNNPR	Kaffa	Gimbo	Gojeb	1311	14
J	2008		4	Amhara	N Gondar	Debark	Dib Bahir	2108	20
K		2 (14–20 months)	19	Amhara	E Gojjam	Basoliben	Michig	2302	20
L			19	Amhara	N Shewa	Ephrata	Laygnaw Ataye	1498	20
M			20	Amhara	N Wollo	Woldia	Gola Menchare	1887	20
N			19	Amhara	S Gondar	Debre Tabor	Hiruy Abaregay	2629	20
P			20	Amhara	S Wollo	Tenta	Watta	2148	20
Q			18	Oromia	Illubabor	Darimu	Tulema	1662	20
R			18	Oromia	Jimma	Seka Chekorsa	Shashemane	1915	20
S			17	SNNPR	Bench Maji	Guraferda	Ottiwa	1147	20
T			18	SNNPR	Kaffa	Gimbo	Chereba	1748	20
U			20	SNNPR	Sheka	Yeki	Depi	1341	20
J	2009		14	Amhara	N Gondar	Debark	Dib Bahir	2108	20
K		3 (26–32 months)	29	Amhara	E Gojjam	Basoliben	Michig	2302	20
L			29	Amhara	N Shewa	Ephrata	Laygnaw Ataye	1498	20
M			30	Amhara	N Wollo	Woldia	Gola Menchare	1887	20
N			28	Amhara	S Gondar	Debre Tabor	Hiruy Abaregay	2629	20
P			27	Amhara	S Wollo	Tenta	Watta	2148	20
Q			30	Oromia	Illubabor	Darimu	Tulema	1662	20
R			30	Oromia	Jimma	Seka Chekorsa	Shashemane	1915	20
S			28	SNNPR	Bench Maji	Guraferda	Ottiwa	1147	20
T			26	SNNPR	Kaffa	Gimbo	Chereba	1748	20
U			32	SNNPR	Sheka	Yeki	Depi	1341	20

*TCC* stands for The Carter Center.

### Physical evaluation – proportion of nets with holes and repairs

**Figure 2:**
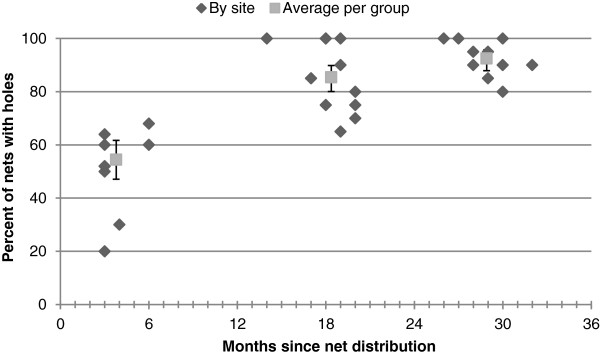
Percentage of nets with any holes, in samples collected at three time periods.

The proportions of nets without damage, with intact seams, and with repairs for each site are shown in Table 2. Among nets in use in the field for three to six months, 86/189 (45.5%) showed no evidence of damage (Figure 2). There were nine (4.8%) with evidence of failed seams and there was no evidence of repairs of either holes or seams. For nets in use in the field for 14 to 20 months, 32/220 (14.6%) showed no evidence of damage (Figure 2). There were 32 (16.0%) nets with evidence of failed seams and evidence of repairs in three nets (1.4%) at 14 to 20 months. At 26 to 32 months, 15/200 (7.5%) had no damage (Figure 2), 17 (8.5%) had failed seams and four nets (2%) were repaired. The difference in proportion of nets with damage at the three time periods was statistically significant (Chi sq 92.9478, p < 0.0001).

**Table 2:** Summary of physical condition of collected nets, by site and time group

**Site**	**Time in potential use (months)**	**No. of nets**	**No. (%) nets undamaged**	**No. (%) nets with seams intact**	**No. (%) nets with repairs**	**No. of holes (≥0.5 cm) per net**		**Hole size (cm)**	
						**Median (IQR)**	**Range**	**Median (IQR)**	**Range**
A	3	25	12 (48)	23 (92)	0	1 (0–2)	0-40	1 (1–2)	0.5-18
B	3	25	12 (48)	24 (96)	0	1 (0–5)	0-13	1 (1–1)	0.5-13
C	3	25	9 (36)	24 (96)	0	1 (0–3)	0-36	1 (1–2)	0.5-10
D	3	25	10 (40)	22 (88)	0	1 (0–6)	0-25	1 (1–2)	0.5-13
E	6	25	10 (40)	24 (96)	0	3 (0–9)	0-46	1 (0.5-2)	0.5-26
F	6	25	8 (32)	24 (96)	0	3 (0–11)	0-28	1 (1–3)	0.5-58
G	3	5	4 (80)	5 (100)	0	0 (0–0)	0-3	1 (0.5-1)	0.5-1
H	3	14	7 (50)	14 (100)	0	0.5 (0–2)	0-30	1 (1–2)	1-5
J	4	20	14 (70)	20 (100)	0	0 (0–3.5)	0-40	1 (1–2)	1-10
	**3-6 mths**	**189**	**86 (46)**	**180 (95)**	**0**	**1 (0–5)**	**0-46**	**1 (1–2)**	**0.5-13**
K	19	20	0 (0)	19 (95)	0	10.5 (4–22)	1-114	1 (1–2)	1-60
L	19	20	2 (10)	19 (95)	1 (5)	16 (3.5-26.5)	0-86	1 (1–3)	1-32
M	20	20	4 (20)	20 (100)	0	4 (1–9)	0-48	1 (1–2)	1-19
N	19	20	7 (35)	18 (90)	0	7 (0–24.5)	0-114	1 (1–2)	1-50
P	20	20	6 (30)	16 (80)	0	6 (0–20)	0-91	1 (1–3)	1-88
Q	18	20	0 (0)	14 (70)	0	59 (25–99.5)	1-263	1 (1–2)	1-120
R	18	20	5 (25)	18 (90)	0	14 (3.5-48.5)	0-144	1 (1–3)	1-155
S	17	20	3 (15)	16 (80)	1 (5)	10.5 (3–18.5)	0-138	1 (1–2)	1-34
T	18	20	0 (0)	13 (65)	0	17 (13–59.5)	1-128	1 (1–2)	1-36
U	20	20	5 (25)	15 (75)	1 (5)	6 (1–37.5)	0-524	2 (1–3)	1-180
J	14	20	0 (0)	19 (95)	1 (5)	14.5 (5.5-36)	1-52	NA	NA
	**14-20 mths**	**220**	**32 (15)**	**187 (85)**	**4 (2)**	**12 (3–36.5**)	**0-524**	1 (1–2)*	1-180*
K	29	20	1 (5)	18 (90)	0	22 (6–30.5)	0-47	NA	NA
L	29	20	3 (15)	20 (100)	1 (5)	10.5 (1–25.5)	0-133	NA	NA
M	30	20	4 (20)	18 (90)	0	5.5 (2.5-20.5)	0-60	NA	NA
N	28	20	1 (5)	20 (100)	1 (5)	11.5 (3.5-27.5)	0-149	NA	NA
P	27	20	0 (0)	18 (90)	0	23.5 (13.5-53)	2-116	NA	NA
Q	30	20	0 (0)	14 (70)	0	77.5 (37.5-142.5)	7-207	NA	NA
R	30	20	2 (10)	19 (95)	0	26.5 (6.5-87.5)	0-236	NA	NA
S	28	20	2 (10)	17 (75)	0	23 (11.5-53)	0-187	NA	NA
T	26	20	0 (0)	19 (95)	1 (5)	66.5 (39.5-131.5)	1-399	NA	NA
U	32	20	2 (10)	20 (100)	1 (5)	18.5 (2–85.4)	0-2690	NA	NA
	**26-32 mths**	**200**	**15 (7.5)**	**183 (92)**	**4 (2)**	**23 (6–55.5)**	**0-2690**	**NA**	**NA**

*excludes site J in 14 to 20 month group (exact hole size not available for nets collected in 2009). *NA* not available.

### Physical evaluation – number of holes

**Figure 3:**
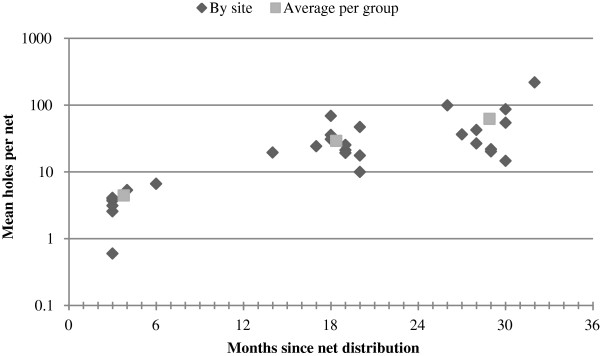
Mean number of holes per net by time group (log scale).

The median and range of the number of holes per net by site are shown in Table 2. The mean holes per net increased from 4.4 (SD 8.4, N = 189) at three to six months to 29.1 (SD 50.1, N = 220) at 14 to 20 months and to 62.2 (SD 205.4, N = 200) at 26 to 32 months (Figure 3). At three to six months, the median number of holes was one (interquartile range [IQR] 0–5, range 0–46) and the median size of the holes was 1.0 cm (IQR 1–2 cm, range 0.5-13 cm) (Table 2). At 14 to 20 months, the median number of holes was 12 (IQR 3–36.5, range 0–524) and the median size of the holes was 1.0 cm (IQR 1–2 cm, range 1–180 cm). At 26 to 32 months, the median number of holes was 23 (IQR 6–55.5, range 0–2690). Exact hole size was not measured at time period 3. The difference in median number of holes at time 1 *versus* time 2 was statistically significant by Wilcoxon signed rank test (z = −9.860, p < 0.0001) and similarly for time 2 *versus* time 3 (z = −3.482, p = 0.0005).

**Figure 4:**
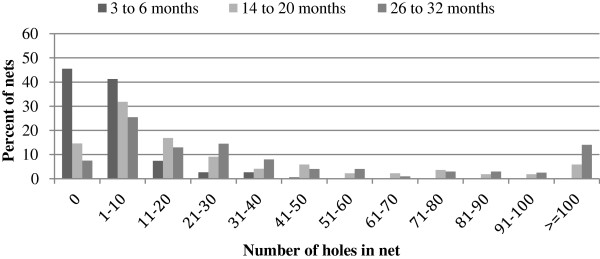
Frequency distribution of number of holes per net, at three time periods.

The distribution of number of holes per net is shown in Figure 4. At three to six months, the highest frequency category was nets with zero holes. At the two subsequent time periods of collection the highest frequency was for nets with one to ten holes. The percentage of nets in this category dropped sharply over the three time periods. The percentage of nets with more than 100 holes was over 10% by time period 3 (26 to 32 months in use).

**Figure 5:**
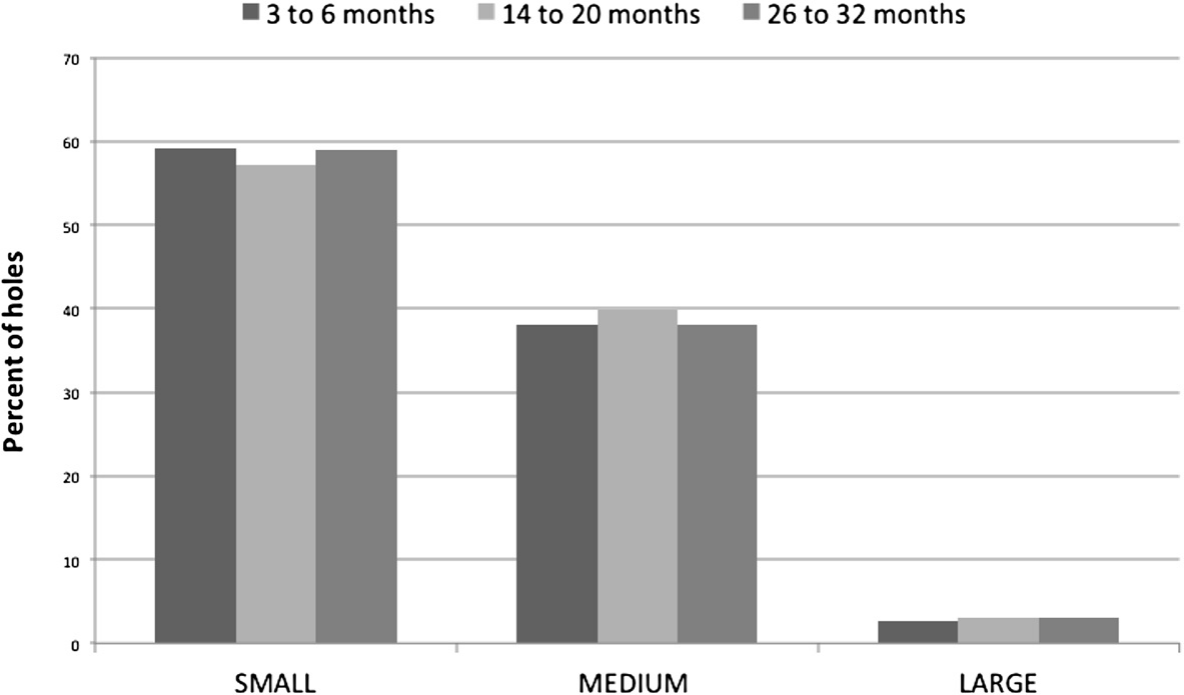
Percentage of holes in each size category, by time group of collection.

Although there were more nets with holes as the time periods progressed, and the number of medium and large holes increased (Table 3), the relative number of holes of different sizes (small <2 cm; medium > =2 and < =10 cm; large >10 cm) did not change between the time periods of collection (Figure 5).

**Table 3:** Summary of hole size distribution and hole index for collected nets

**Site**	**Time in potential use (months)**	**No. of nets**	**Mean no small holes per net**	**Mean no medium holes per net**	**Mean no large holes per net**	**Mean proportionate hole index per net**
A	3	25	2.2	1.7	0.1	24.4
B	3	25	2.0	0.6	0.04	9.2
C	3	25	1.6	1.4	0.04	16.8
D	3	25	2.2	1.7	0.1	21.8
E	6	25	4.2	2.2	0.2	37.8
F	6	25	3.6	2.8	0.3	44.4
G	3	5	0.6	0	0	0.6
H	3	14	2.7	1.0	0	11.7
J	4	20	3.1	2.2	0.1	28.5
	**3-6 months**	**189**	**2.6**	**1.7**	**0.1**	**24.3**
K	19	20	16.3	8.3	0.8	135.8
L	19	20	11.0	7.8	0.6	114.3
M	20	20	6.2	3.6	0.2	49.8
N	19	20	14.7	5.9	0.7	104.2
P	20	20	9.5	7.3	0.8	120.0
Q	18	20	38.8	28.7	1.5	381.1
R	18	20	16.6	13.2	1.2	202.2
S	17	20	18.0	5.8	0.5	95.4
T	18	20	23.4	11.9	0.5	155.7
U	20	20	18.6	26.4	2.1	373.3
J	14	20	10.0	8.9	0.7	128.9
	**14-20 months**	**220**	**16.6**	**11.6**	**0.9**	**169.1**
K	29	20	10.0	9.8	0.6	128.6
L	29	20	13.6	7.6	0.7	120.8
M	30	20	6.5	7.1	1.0	126.4
N	28	20	12.3	13.6	0.8	179.5
P	27	20	18.6	16.0	1.9	266.2
Q	30	20	47.7	37.0	2.3	509.5
R	30	20	29.9	22.5	2.0	341.6
S	28	20	22.0	18.5	2.0	297.2
T	26	20	62.1	34.1	2.9	531.4
U	32	20	143.7	71.1	4.4	1026.8
	**26-32 months**	**200**	**36.6**	**23.7**	**1.8**	**352.8**

### Physical evaluation – location of holes

**Figure 6:**
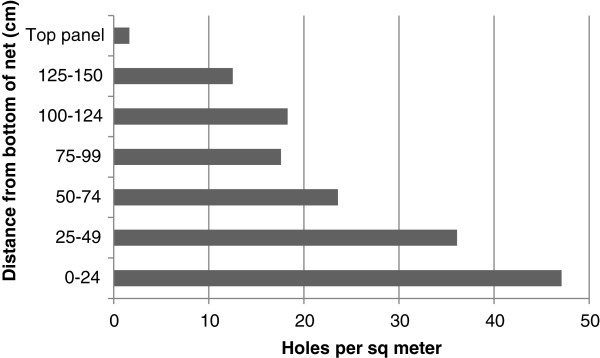
**Frequency distribution of holes by location on net, for nets collected after 14 to 20 months of use from sites H through U.** Frequency normalized according to the area of the net represented.

The location of the holes on the net is shown in Figure 6 for the 14 to 20 month group of nets collected in 2008 from ten sites. The numbers of holes were standardized to holes per square metre because of the varying area of the top and side panels evaluated. Most of the holes (58.7% of 6,005 holes) and the greatest hole density were in the lower half (75 cm) of the side panels, although holes were recorded at all heights of the side panel, as well as in the top panel. Results were similar for the three to six month group. Location of holes was not noted in the 26 to 32 month time group 3.

### Hole index

Mean hole index by site is given in Table 3. Proportionate hole index per net ranged from none to 11,881. By site it ranged from 0.6 (in site G, time group 1) to 1,026.8 (in site U, time group 3). The mean hole index by time group increased from 24.3 at three to six months, to 169.1 at 14 to 20 months and to 352.8 at 26 to 32 months. Although a somewhat arbitrary number based on relative area of holes in different size categories, the hole index attempts to capture the increase in physical damage over the time period in a single measure.

Table 4 shows the classification of hole index into 4 categories following Kilian *et al.*[10]. At 3 to 6 months, the majority of the nets (78.3%) were classified as “good”, but this had decreased to 20.5% at 26 to 32 months. The percentage of nets classified as “poor” increased from 0% at 3 to six months to 30.0% at 26 to 32 months. Therefore almost one third of the nets were essentially unusable by this time. The percentages classified as either “mediocre” or “poor” were 3%, 28% and 45% at the three time periods respectively.

**Table 4:** Categorization of nets by hole index

	**Number and percentage of nets by hole index category**				
**Time in potential use (months)**	**Good <25**	**Fair 25-174**	**Mediocre 175-299**	**Poor > =300**	**Total**
3-6 months	148	35	6	0	189
	*78*.*3*	*18*.*5*	*3*.*2*	*0*.*0*	
14-20 months	71	88	27	34	220
	*32*.*3*	*40*.*0*	*12*.*3*	*15*.*5*	
26-32 months	41	70	29	60	200
	*20*.*5*	*35*.*0*	*14*.*5*	*30*.*0*	
Total	260	193	62	94	609
	*42*.*7*	*31*.*7*	*10*.*2*	*15.4*	

## Discussion

This investigation was unusual in that net evaluation began very shortly after distribution. Since LLINs are expected to last for multiple years, most prior studies have been of nets a year or more after distribution. However, for programme planners preparing for replacement campaigns, it would be useful to have meaningful data about nets in the field and their rate of deterioration as early as possible. Results from this study showed that in as little as three to six months, a quantifiable picture began to emerge regarding the physical deterioration of nets in the field. Future LLIN monitoring efforts can therefore start less than a year after distribution in order to give planners a head start on developing timetables for replacement.

Although some nets were still in good physical condition even after a year-and-a-half in this study, a substantial proportion showed significant deterioration: 68% had holes and 28% were classed as ‘mediocre’ or ‘poor’ by hole index at 14 to 20 months of use. The distribution of holes per net was highly skewed, with a few nets having many holes and most nets with few holes. For this reason, statistical descriptions of overall condition of a group of nets are better described by the median and not the mean number of holes, the latter being disproportionately impacted by the existence of a few heavily damaged nets. However, the size of holes must be taken into account as well: one large hole may be as problematic (or even more so) than a large number of small holes, since large holes can let more mosquitoes inside of the net without contacting the insecticidal fibres.

The two schemes proposed [9,10] to estimate a standardized hole index differ in both the number of size categories and the relative factors applied to each. In the first scheme, WHO [9] recommended four size categories of 0.5 to 2 cm, 2 to 10 cm, 10 to 25 cm and >25 cm, with midpoint hole diameter of 1.25, 6, 17.5 and 30 cm respectively. The relative multiplication factors based on corresponding hole areas for these groups are 1, 23, 196 and 578. Secondly, Kilian *et al.*[10] and Batisso *et al.*[11] used three size categories <2 cm, 2 to 10 cm and >10 cm (finger, fist and head). They estimated average hole areas to be 4, 36 and 225 sq cm respectively, giving multiplication factors of 1, 9 and 56 for small, medium and large holes to arrive at the proportionate hole index. The current study commenced before either of these schemes was published, but elected to use the method of Kilian *et al*. [10] and Batisso *et al*. [11] for hole index calculation as it is based on three size categories similar to those used here, and to enhance comparability between studies in Ethiopia.

A limitation of this study is that net attrition (complete loss of nets through disposal, diversion to other use, sale, or donation to others outside the household) was not measured, so net deterioration and loss may have been underestimated. Attrition has been estimated to be as high as 32% over three years (Batisso *et al*. [11]). Other limitations include the convenience sampling method for the sites and households, and the fact that the sites were at a range of altitudes, so net use (and as a result, net wear) may differ significantly depending on mosquito populations and perceived risk of malaria.

Repairs to nets that had developed holes were rarely observed in this study. Low repair rates were also reported by Smith *et al*. [6], Kilian *et al*. [10] and Shirayama *et al*. [12] so this appears to be a widespread issue. However, Bhatt *et al*. [13] found a total of 750 repairs, or average 1 repair per LLIN, in the forms of stitches (63.9%), knots (35.8%) and patches (0.3%) in their evaluation of Interceptor® LLINs (n = 932) in central India. It is likely that longevity of nets can be significantly improved simply by making repairs to them and perhaps by extension, encouraging behaviour that would prevent the nets from developing holes to start with.

Although the cause of the holes in the net studied here is not known, household observations suggested that fire embers together with damage from animals and sharp edges on beds or mats were major contributors. This will be explored further in risk factor analysis, using hole index and insecticide concentration as outcomes, for the 2008 and 2009 collections in a subsequent paper. Holes were observed on all sides of the net including the top panel, indicating that all parts of the nets are susceptible to damage, but net density was greater in the lower sides of the net. Of note, the relative proportion of small, medium and large holes did not change over time. Thus the hole size (and hole index) distribution at a future date could possibly be predicted using data from nets used for only a short period. Furthermore, if the proportion of small, medium and large holes is different for different net brands, it could become a measure of net quality (quality increasing as large:small hole ratio decreases). Knowing the ratio of large to small holes could have a profound impact on program planning and procurement decisions, and it would be desirable for future similar durability studies to report on this ratio.

Comparison of the physical deterioration rates between studies is difficult because of differing methodologies and time periods of assessment. Kilian *et al*. [10] observed that for PermaNets in Uganda, >70% had holes in them after one year (i e, <30% were undamaged). In this study, net attrition was not measured, as in the current study. Although nets were not collected after exactly one year, the findings in the current study were fairly consistent: it was observed that 54.5% of nets were damaged after three to six months and 85.5% after 14 to 20 months. In Tanzania, Maxwell *et al*. [14] defined “intact” nets as having <20 holes <2 cm in diameter, <5 holes 2–5 cm in diameter, and <2 holes >5 cm in diameter. Maxwell *et al*. found that 84.4% of Olyset nets in highland villages were ‘intact’ after four to five years and 38.4% of the nets in lowland villages were ‘intact’ after six to seven years [14]. In the current study, the time period is shorter and the hole size comparison is not strictly similar. However, it was found that 65.5% of nets had <20 holes (any size) at 14 to 20 months and 46.8% had <20 holes at 26 to 32 months, suggesting that these PermaNet®2.0 were deteriorating more quickly than the Olyset nets observed in Maxwell’s study.

In Chad, Allan *et al*. [15] used a proportionate hole index to assess LLIN durability in Interceptor, Olyset, and PermaNet after one year of household use, and assessed damage as holes < 2 cm, >2-5 cm and >5 cm. A total of 876 LLINS were assessed and 25% had a hole index greater than 300; these nets were classified in their study as “unserviceable and irreparable”. The largest percentage of nets (i.e. 44.5%), classified as “in partial service” had proportionate hole indices in the middle ranges of 25–174 and 175-299 [15]. The most relevant comparison for our study is to that of Batisso *et al*. [11] in Ethiopia, who observed that after three years, 70% of nets were still in ‘good’ or ‘fair’ condition as defined by a hole index of <175, whereas in this study the percentage overall in these two categories was lower at a slightly earlier time period: 50.5% at 26 to 32 months. As noted by Batisso *et al*., work still needs to be done to determine when nets should be classified as ‘unserviceable’, as well as comparisons of net life in different circumstances within the same country and between countries.

## Conclusions

The current study provides a description of the deterioration of PermaNet®2.0 LLINs distributed in Ethiopia with support from The Carter Center in 2007. Many nets were physically damaged after only three to six months’ use (54.5%, ranging from 20-68% by site), which increased to 85% on average (range 65-100% by site) after 14 to 20 months and to 93% (range 80-100% by site) after 26 to 32 months. The condition of nets was skewed, with fewer nets being heavily damaged and many nets showing relatively light damage. The mean hole index was 24.3, 169.1 and 352.8 at the three time periods respectively. An almost complete lack of repairs suggests that a programme of teaching and encouraging net repairs may be a means of increasing LLIN longevity. Using the hole index, almost one third of the nets still present in households were classed as unusable and ineffective after two and a half years of potential use.

## Competing interests

The authors declare that they have no competing interests.

## Authors’ contributions

The study was planned by PME, FOR, PMG and TG. ABW, SS, GYA and MD collected nets from the field with assistance from EBS, TE and TG. ABW and GYA conducted the hole assessments and data management. ABW, PMG, AEP and YBT did data analysis. AWM did the mapping. PMG, ABW and AEP wrote the paper, which was reviewed by all authors. All authors read and approved the final manuscript.
